# Cardiac ECV is more robust than post-contrast cardiac T_1 _for evaluating temporal changes in LV fibrosis

**DOI:** 10.1186/1532-429X-16-S1-P25

**Published:** 2014-01-16

**Authors:** Kyungpyo Hong, Matthias Koopmann, Eugene G Kholmovski, Eric C Huang, Nan Hu, Richard Levenson, Sathya Vijayakumar, Derek J Dosdall, Ravi Ranjan, Daniel Kim

**Affiliations:** 1 UCAIR, Radiology, University of Utah, Salt Lake City, Utah, USA; 2 CARMA Center, University of Utah, Salt Lake City, Utah, USA; 3 Division of Cardiology, Internal Medicine, University of Utah, Salt Lake City, Utah, USA; 4 Department of Pathology and Laboratory Medicine, University of California, Davis Medical Center, Sacramento, California, USA; 5 Division of Epidemiology, Internal Medicine, University of Utah, Salt Lake City, Utah, USA

## Background

Post-contrast cardiac T_1 _measurement has been reported to be correlated with interstitial fibrosis burden. However, post-contrast cardiac T_1 _can also be influenced by a variety of confounders, including: cardiac function, renal function, hematocrit, magnetic field strength, contrast agent type and dosage, and specific delayed imaging time after contrast agent administration. To compensate for these confounders, investigators have proposed to measure extracellular volume (ECV). Despite the advantages of ECV over cardiac T_1_, systematic studies comparing the two measurements are lacking [1]. The purpose of this study was to compare the effectiveness of post-contrast cardiac T_1 _and ECV for evaluating the temporal changes in left ventricular (LV) fibrosis in an established canine model with chronic atrial fibrillation (AF)[2].

## Methods

Seventeen mongrel dogs with different durations (0-22 months) of chronic AF were scanned multiple times for a total of 46 CMR scans at 3T (Verio, Siemens). Cardiac T_1 _maps were acquired in 3 short-axis planes (base, mid, and apex) using the arrhythmia-insensitive-rapid (AIR) cardiac T_1 _mapping pulse sequence [3] based on B_1_-insensitive saturation-recovery of magnetization preparation, with the following relevant imaging parameters: spatial resolution = 1.4 × 1.4 × 7.0 mm, temporal resolution = 217 ms, saturation-recovery time = 600 ms. Cardiac T_1 _maps were acquired pre-contrast and 15 min after a bolus injection of Gd-BOPTA (MultiHance; 0.15 mmol/kg). Blood samples were drawn during MRI for hematocrit calculation. For image analysis, myocardial contours and blood pool were manually segmented, and T_1 _and ECV values were calculated. LV ejection fraction (LVEF) was calculated using cine MRI. Temporal changes in post-contrast LVEF, LV T_1_, blood T_1_, and ECV were modeled with linear mixed effect models to account for repeated measurements over disease duration. Four animals were sacrificed at different durations of AF (0-22.6 months) for histologic quantification of LV fibrosis.

## Results

**Figure 1:**
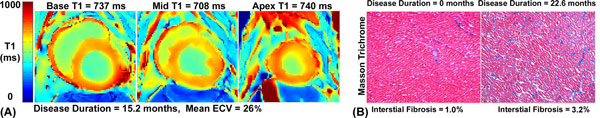
**(A) Representative post-contrast AIR cardiac T_1 _maps of a dog with AF duration = 15.2 months**: (left) basal (T_1 _= 737 ms), (middle), mid-ventricular (T^1 ^= 708 ms), and (right) apical (T_1 _= 740 ms) planes, with mean ECV = 26%. (B) Histologic evaluation of Masson's trichrome staining of LV tissue (midwall) samples from the antero-lateral LV in different dogs sacrificed at different durations of AF: (left) baseline and (right) 22.6 months. All specimens displayed with 10× magnification.

**Figure 2:**
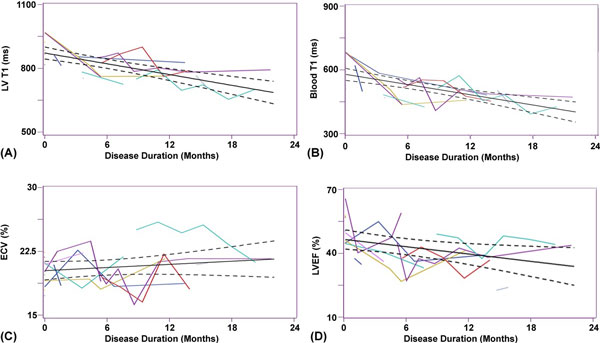
**Plot of the estimated regression line (solid line), along with the 95% confidence intervals (dashed lines), describing the temporal changes in: (A) LV T_1_, (B) blood T_1_, (C) ECV, and (D) LVEF**. All four parameters, except ECV, changed significantly with disease duration over 22 months. Different colors represent individual temporal trends.

Figure 1 shows post-contrast cardiac T_1 _maps of a dog with disease duration = 15.2 months, as well as LV tissue samples with Masson's trichrome staining at baseline (interstitial fibrosis = 1.0%) and 22.6 months of AF duration (interstitial fibrosis = 3.2%). As shown in Figure 2, all four parameters (p < 0.05), except ECV (p = 0.29), changed significantly with disease duration of 22 months. Note that the temporal trends in LV and blood T_1 _are similar. Compared with histologic quantification of fibrosis and extracellular space, ECV agreed better than LV T_1_.

## Conclusions

This study suggests that ECV is a more robust measure of extracellular space than post-contrast LV T_1_, especially for evaluating temporal changes in LV fibrosis.

## Funding

Ben B. and Iris M. Margolis Foundation.
